# Outcomes of Patients With Surgically and Pathologically Staged IIIA-IVB Pure Endometrioid-type Endometrial Cancer: A Taiwanese Gynecology Oncology Group (TGOG–2005) Retrospective Cohort Study (A STROBE-Compliant Article)

**DOI:** 10.1097/MD.0000000000003330

**Published:** 2016-04-18

**Authors:** Jen-Ruei Chen, Ting-Chang Chang, Hung-Chun Fu, Hei-Yu Lau, I.-Hui Chen, Yu-Min Ke, Yu-Ling Liang, An-Jen Chiang, Chia-Yen Huang, Yu-Chieh Chen, Mun-Kun Hong, Yu-Chi Wang, Kuo-Feng Huang, Sheng-Mou Hsiao, Peng-Hui Wang

**Affiliations:** From the Department of Obstetrics and Gynecology, MacKay Memorial Hospital and MacKay Junior College of Medicine, Nursing and Management, Taipei (J-RC); Department of Obstetrics and Gynecology, Chang Gung Memorial Hospital and Chang Gung University, Taoyuan (T-CC); Department of Obstetrics and Gynecology, Kaohsiung Chang Gung Memorial Hospital and Chang Gung University, Kaohsiung (H-CF); Department of Obstetrics and Gynecology, Taipei Veterans General Hospital, Taipei (H-YL, P-HW); Department of Obstetrics and Gynecology, National Taiwan University Hospital, Hsin-Chu Branch, Hsinchu (I-HC), Department of Obstetrics and Gynecology, Taichung Veterans General Hospital, Taichung (Y-MK), Department of Obstetrics and Gynecology, National Cheng Kung University Hospital, National Cheng Kung University, Tainan (Y-LL); Department of Obstetrics and Gynecology, Kaohsiung Veterans General Hospital, Kaohsiung (A-JC); Department of Obstetrics and Gynecology, Cathay General Hospital, Taipei (C-YH); Graduate Institute of Clinical Medicine, College of Medicine, National Taiwan University, Taipei, Taiwan (C-YH); Department of Obstetrics and Gynecology, Kaohsiung Medical University Chung-Ho Memorial Hospital and Kaohsiung Medical University, Kaohsiung (Y-CC), Department of Obstetrics and Gynecology, Buddhist Tzu Chi General Hospital and Tzu Chi University, Hualien (M-KH), Department of Obstetrics and Gynecology, Tri-Service General Hospital and National Defense Medical Center (Y-CW); Department of Obstetrics and Gynecology, Chi-Mei Medical Center, Tainan (K-FH); Department of Obstetrics and Gynecology, Far Eastern Memorial Hospital, New Taipei City (S-MH); Department of Obstetrics and Gynecology, National Yang-Ming University, Taipei (H-YL, P-HW); and Department of Medical Research, China Medical University Hospital, Taichung (P-HW), Taiwan.

## Abstract

In the management of patients with advanced-stage pure endometrioid-type endometrial cancer (E-EC), such as positive lymph nodes (stage III) or stage IV, treatment options are severely limited. This article aims to investigate the outcome of women with FIGO III-IV E-EC (based on FIGO 2009 system).

The retrospective cohort study, based on the Taiwanese Gynecologic Oncology Group (TGOG-2005), enrolled patients undergoing staging surgery to have a pathologically confirmed FIGO III-IV E-EC from 22-member hospitals between 1991 and 2010.

This cohort included 541 patients (stage III, n = 464; stage IV, n = 77). Five-year overall survival (OS) was 70.4%. Median progression-free survival (PFS) was 43 months (range 0–258 months) and median OS was 52 months (range 1–258 months). Multivariate analysis showed that FIGO stage, >1/2 myometrial invasion (hazard ratio [HR] 1.53, 95% confidence interval [CI] 1.12–2.09; *P* = 0.007), histological grade 3 (HR 2.0, 95% CI 1.47–2.75; *P* < 0.001), and metastases of pelvic and para-aortic lymph nodes (PLN and PALN) (HR 2.75, 95% CI 1.13–6.72; *P* < 0.001) were independent risk factors for PFS. FIGO stage, >1/2 myometrial invasion (HR 1.89, 95% CI 1.34–2.64; *P* < 0.001), and histological grade 3 (HR 2.42, 95% CI 1.75–3.35; *P* < 0.001) influenced OS. Complete dissection of PLN and PALN (HR 0.27, 95% CI 0.16–0.45; *P* < 0.001, and HR 0.14, 95% CI 0.08–0.26; *P* < 0.001) and the following paclitaxel-based therapy (HR 0.61, 95% CI 0.79–0.92; *P* = 0.017, and HR 0.48; 95% CI 0.31–0.75; *P* = 0.001) provided the better PFS and OS, respectively.

In management of women with FIGO III-V E-EC, combination of complete staging surgery (complete dissection of PLN and PALN is included) and the following paclitaxel-based therapy could provide the better chance to survive. Patients with tumor >1/2 myometrial invasion and histological grade 3 are risky for disease-related mortality.

## INTRODUCTION

Endometrial cancer (EC) is the most common gynecologic malignancy in Europe and North America,^1^ and this trend is also found in Taiwan.^2^ The incidence rate of EC doubled in the past decade.^2^ Traditional classification of EC is based on clinical and endocrine features, including type I endometrioid (E-EC) and type II non-endometrioid type^1^; the former contributes to the majority of cases and is often diagnosed at the FIGO (International Federation of Gynecology and Obstetrics) I stage.^1^ By contrast, <20% of EC patients having tumor spreads to pelvic lymph nodes (PLNs) and para-aortic lymph nodes (PALNs) or distant sites (FIGO III-IV)^3^ were associated with poor outcome, and presented as a therapeutic challenge.^4^ There is a pocket of studies addressing the outcome for the FIGO III-IV EC, but there were many limitations in these studies, including a small fraction of E-EC in enrolled EC patients,^3,5,6^ and a small sample size of E-EC, contributing to uncertainty.^6–8^ It is believed that staging surgery is a main treatment for all E-EC; however, the extent of surgery (complete or incomplete staging surgery) and the following postoperative adjuvant therapy are still debated.^6–17^

In theory, localized tumor can be managed by surgery and/or radiation therapy (RT) with/without chemotherapy (CT), but distant or widespread dissemination needs CT. For FIGO III-IV E-EC patients, CT might be needed because of high possibility of widespread dissemination.^18^ A phase III Gynecologic Oncology Group (GOG) study showed the benefits of CT in FIGO III-V EC patients, based on the findings of a better 5-year overall stage-adjusted survival in CT group compared with RT (50% vs 38%).^3^ A Cochrane Systematic Review found the benefits of CT, although evidence is moderate.^10^ The American Society of Clinical Oncology (ASCO) clinical practice guideline recommended that CT played an important role in FIGO III-IV EC patients.^19^ By contrast, not all studies supported the benefits of CT. One report from Mayo Clinic, USA, did not find the survival benefits in patients treated with CT (43% [CT] vs 42% [non-CT]).^20^ Furthermore, other concern of CT on the increase of toxicity is raised by Cochrane Systematic Review because more adverse effects were found in the CT group compared with those in the RT group.^10^

With inconsistent results of CT, some suggested that combination therapy and/or multimodality strategy might be another choice.^7,13–15^ However, there is no agreement about the sequence of CT or RT, as well as the regimen of CT.^8,10,18,21,22^ To investigate the outcome of the patients with advanced-stage E-EC and clarify the role of postoperative adjuvant therapy for these patients, the Taiwanese Gynecologic Oncology Group (TGOG) conducted this study.

## MATERIAL AND METHODS

This multicenter retrospective study was conducted by the TGOG (TGOG-2005) and the institutional review board (IRB) approvals were gotten from all studied sites. The criteria for enrollment included: pure E-EC; completely staging surgery (cytology, total hysterectomy, bilateral salpingo-oophorectomy [BSO], dissection and/or sampling of PLN [PLND and/or PLNS], dissection and/or sampling of PALN [PALND and/or PALNS], and tumor excision, and/or omentectomy) to confirm the FIGO III-IV pathologically, based on FIGO 2009 system; and the study period between January 1991 and December 2010.

The clinical follow-up included pelvic and physical examinations, vaginal cytology, tumor markers, and imaging examinations when clinically indicated. Recurrence of disease was defined as evidence of image study based on the modified response evaluation criteria in solid tumors (mRECIST) criteria and/or pathology/cytology confirmation. The sites of recurrence were classified as local (inside the true pelvis and para-aortic lymphatic region), distant (outside pelvis, upper abdominal organs, chest/mediastinal lymphatic region, skin or brain) or both. The aim of the present study was to investigate the primary outcome of women with FIGO III-IV E-EC after initial treatment. We did not analyze the treatment of women when the recurrent diseases were detected.

Progression-free survival (PFS) was defined as the interval between the primary surgery and the date of confirmed recurrence or disease progression or disease-free status on the medical records. Overall survival (OS) was calculated to the date of disease-related death or last follow-up. Disease-related death was defined as death caused directly by the disease itself or indirectly by disease-associated complications and treatment. Cases lost during the follow-up and those alive at the end of the follow-up period were considered censored observations. Survival curves were generated using the Kaplan–Meier method, and the differences between survival curves were calculated using the log-rank test. Cox proportional hazard methods were used to evaluate prognostic factors for survival. Multivariate analysis using Cox stepwise forward regression was conducted for the covariate selected in univariate analysis with a *P* value <0.05. Hazard ratio (HR) and 95% confidence interval (CI) were calculated using the Wald test. A *P* value >0.05 was considered to be statistically significant. All statistical analyses were conducted with SAS version 9.3 (SAS Institute, Cary, NC) and Stata Statistical Software, version 12.0 (Stata Corporation, College Station, TX).

## RESULTS

Five hundred and forty-one patients met the inclusion criteria. The characteristic of all patients is shown in the Table 1. The median age was 53 years, and the median body mass index (BMI) was 25.3 kg/m^2^. There were 38.3% of enrolled patients (n = 207) treated with PLNS and PALNS and only 120 women (22.2%) had both LNDs (PLND and PALND). Twenty-six patients (4.8%) in the present study had isolated metastases of PALN without concomitant metastases of PLN. Nearly all patients were treated with postoperative adjuvant therapy, such as CT, RT, or both after a complete surgery. The therapeutic protocols of these adjuvant therapy varied greatly, including others (not specified, absence of CT, and/or RT), no CT, no RT, CT alone, RT alone, sequential CT then RT (CT→RT), sequential RT then CT (RT→CT), sandwich setting of CT and RT (CT→RT→CT), reverse sandwich setting (RT→CT→RT), and concomitant CT and RT.

**TABLE 1:**
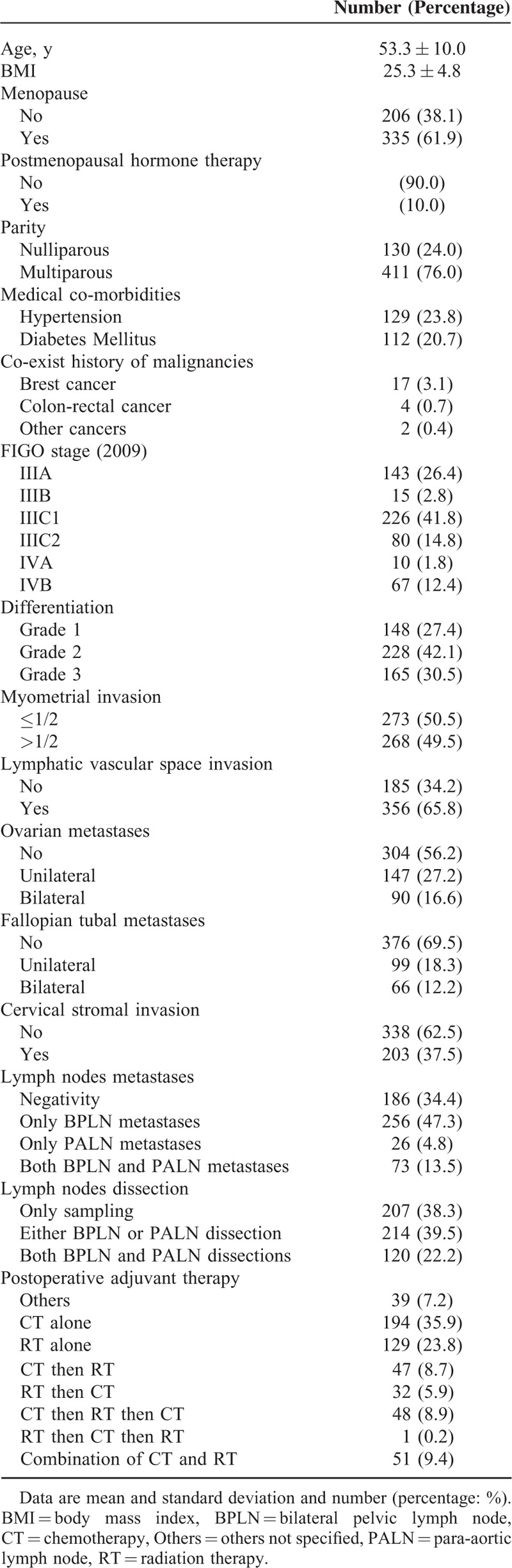
Characteristic of the Enrolled Patients

During the end of the follow-up, a total of 174 patients (32.2%) had a recurrent disease. Among the patients with recurrence, 47 (27.0%) had a local recurrence, 79 patients (45.4%) had a distant recurrence, and 48 patients (27.5%) had both. The median PFS of all patients was 43 months, ranging from 0 to 258 months. Five-year OS was 85.3% for IIIA, 66.7% for IIIB, 71.7% for IIIC1, 66.3% for IIIC2, 50% for IVA, and 43.3% for IVB, contributing to the 70.4% of OS and 52 months of the median OS of all patients (from 1 to 258 months).

**FIGURE 1:**
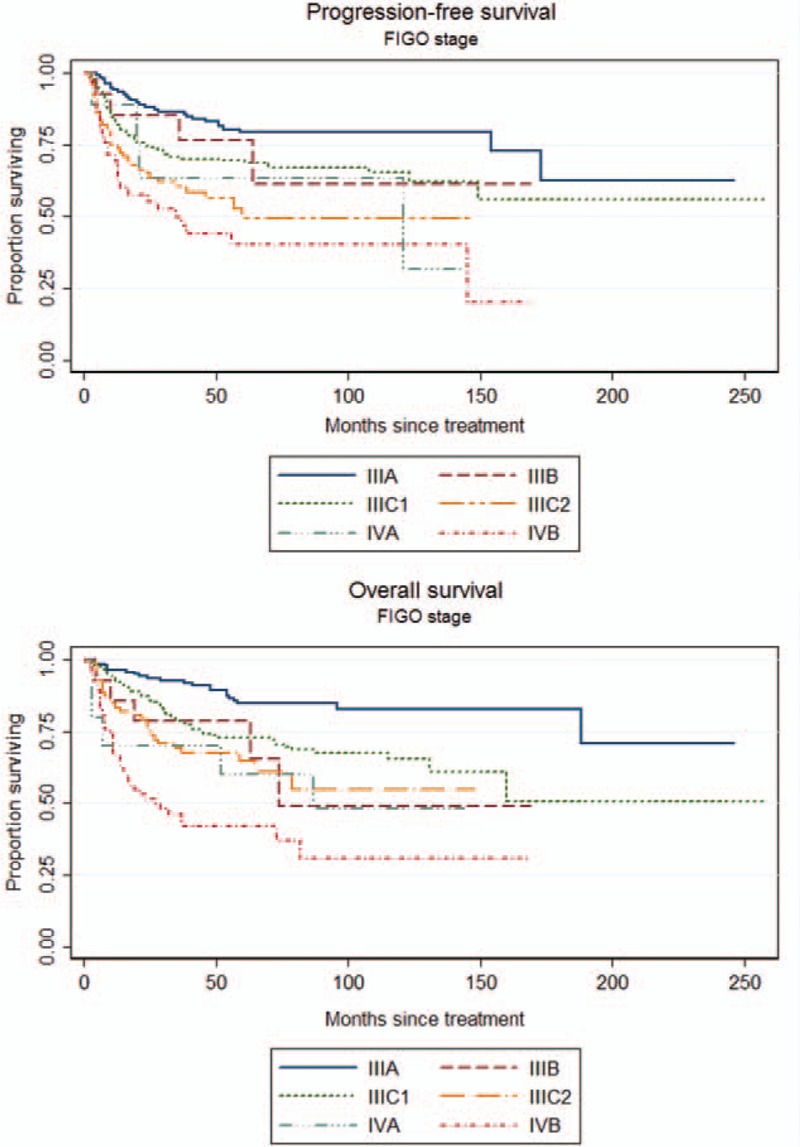
Progression-free survival (PFS) and overall survival (OS) curves according to the FIGO stage (PFS: log-rank test: *P* < 0.001; OS: log-rank test: *P* < 0.001).

**FIGURE 2:**
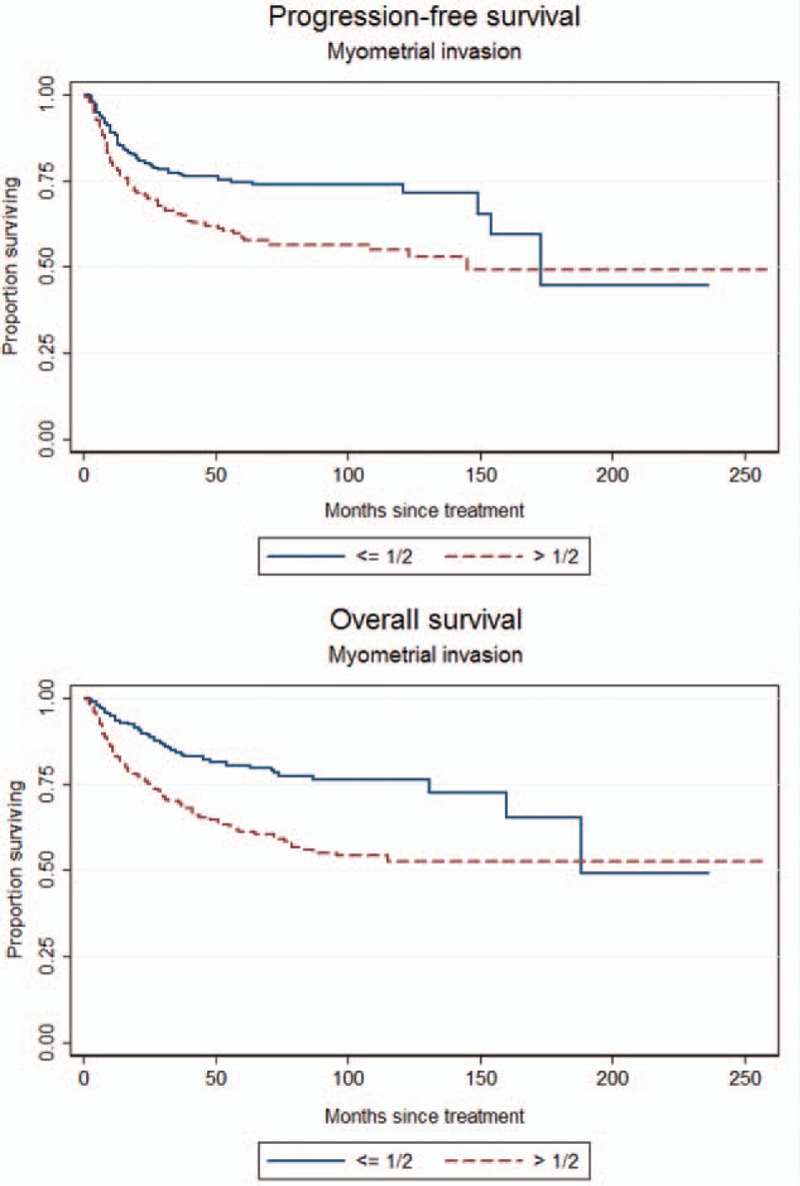
Progression-free survival (PFS) and overall survival (OS) curves according to the status of myometrial invasion (PFS: 56.0 vs 34.5 months, log-rank test: *P* < 0.001; OS: 59.0 vs 41.5 months, log-rank test: *P* < 0.001).

**FIGURE 3:**
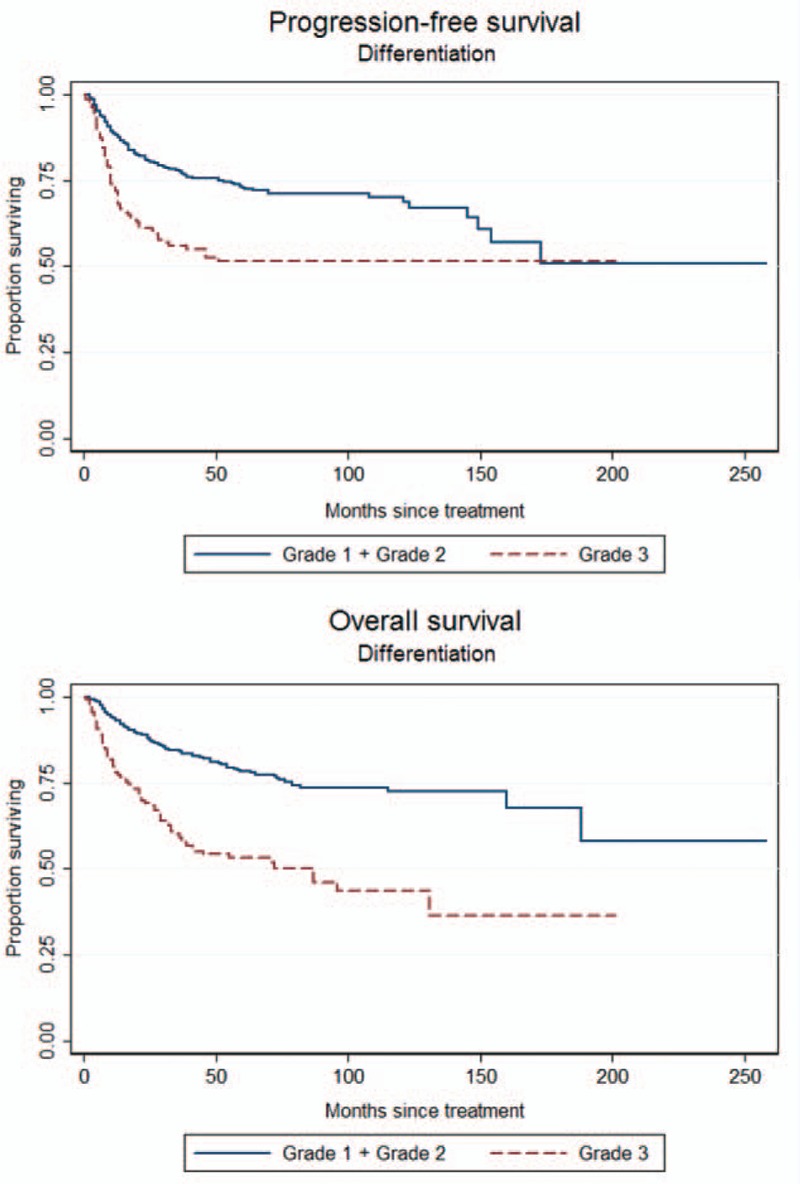
Progression-free survival (PFS) and overall survival (OS) curves according to the status of cell differentiation (PFS: 57 vs 20 months, log-rank test: *P* < 0.001; OS: 61 vs 31 months, log-rank test: *P* < 0.001).

**FIGURE 4:**
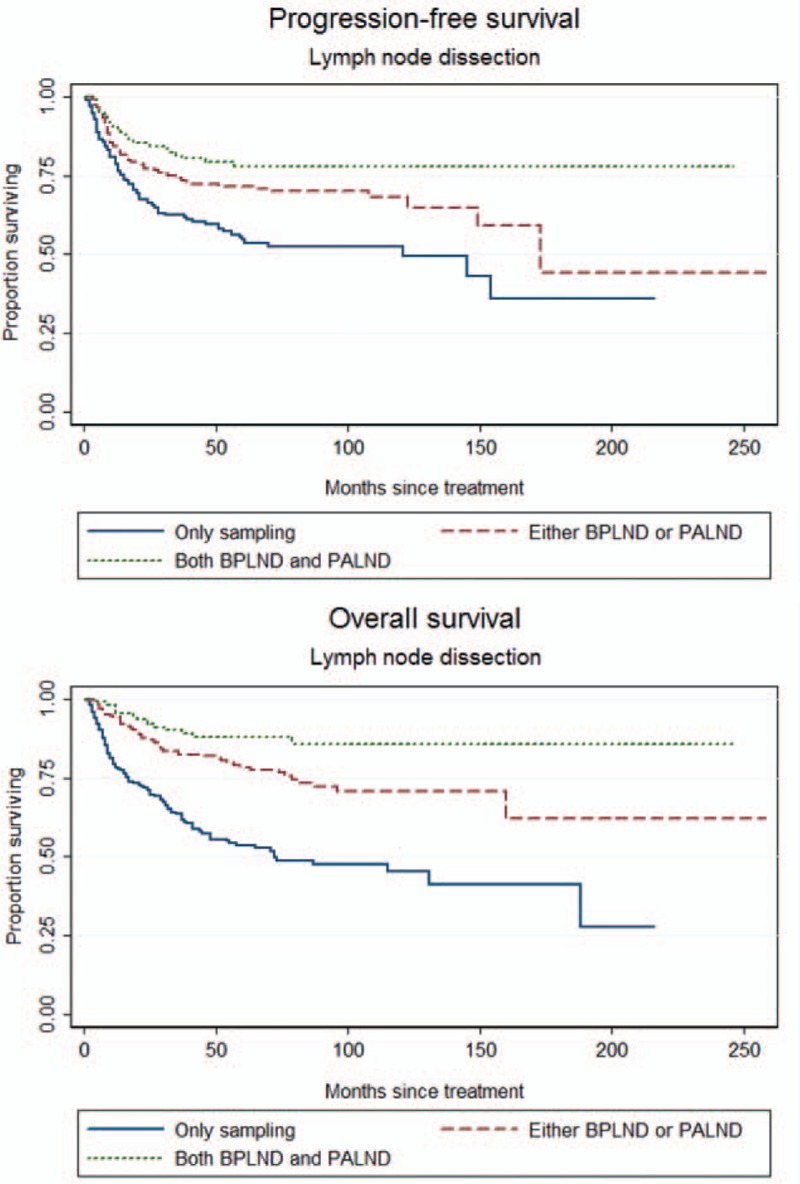
Progression-free survival (PFS) and overall survival (OS) curves according to the status of lymph-node dissection, including bilateral pelvic and para-aortic area (BPLND and PALND; PFS: log-rank test: *P* < 0.001 and OS: log-rank test: *P* < 0.001).

To identify which factors would contribute to the PFS, univariate analysis was performed. As shown in the Table 2, age >53 years, slim patients, FIGO stage, >1/2 myometrial invasion, histological grade 3 (poor differentiation), and LN metastases worsen the PFS of the patients. The worst outcome was found at the FIGO stage IIIC2 and IVB with the median PFS was 28.5 months and 10 months, respectively (Figure 1). The median PFS of the other FIGO stages included 65 months in FIGO stage IIIA, 39 months in FIGO stage IIIB, 50 months in FIGO stage IIIC1, and 38 months in FIGO stage IVA. Patients with >1/2 myometrial invasion had the worse outcome than those with ≤1/2 myometrial invasion (Figure 2). Patients with histological grade 3 had the worse prognosis than those with grades 1 or 2 (Figure 3). Patients who were treated with LND (either PLND or PALND) had a significantly better prognosis than those patients who were not, and patients undergoing both LNDs had a best chance to survive (Figure 4).

**TABLE 2:**
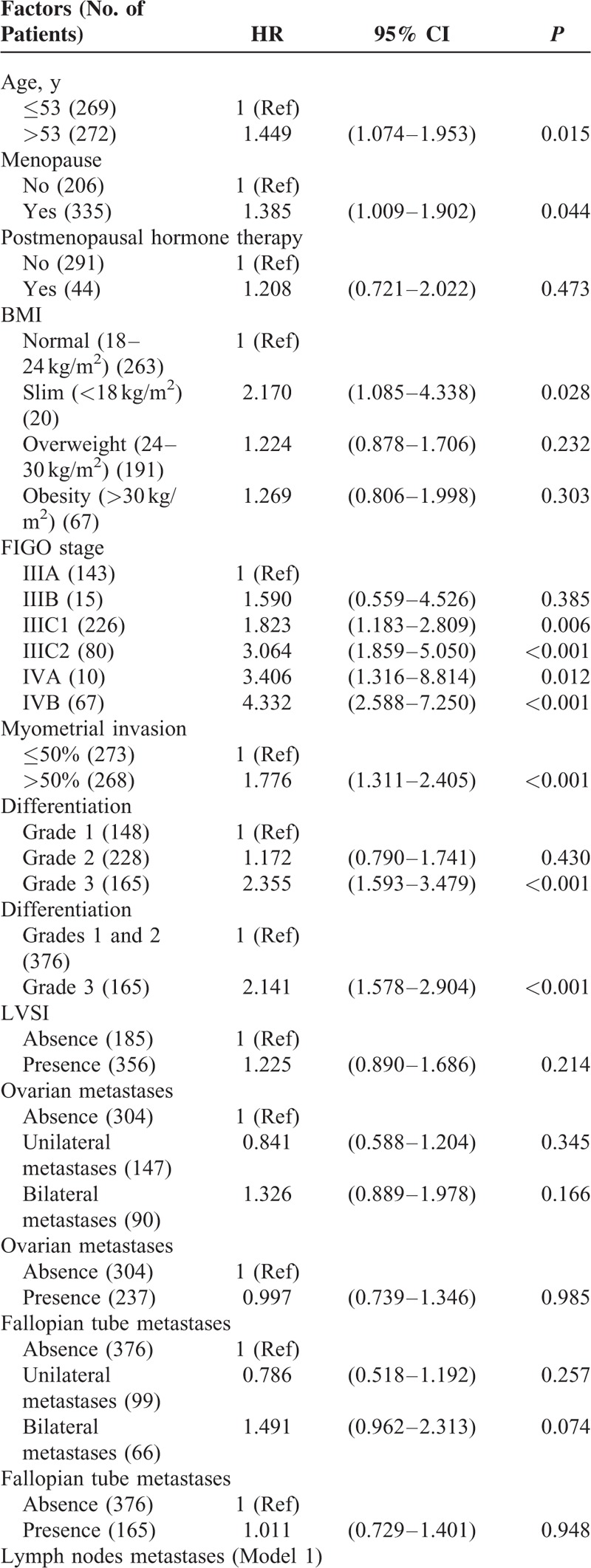
Univariate Analysis for Progress-free Survival

**TABLE 2 (Continued):**
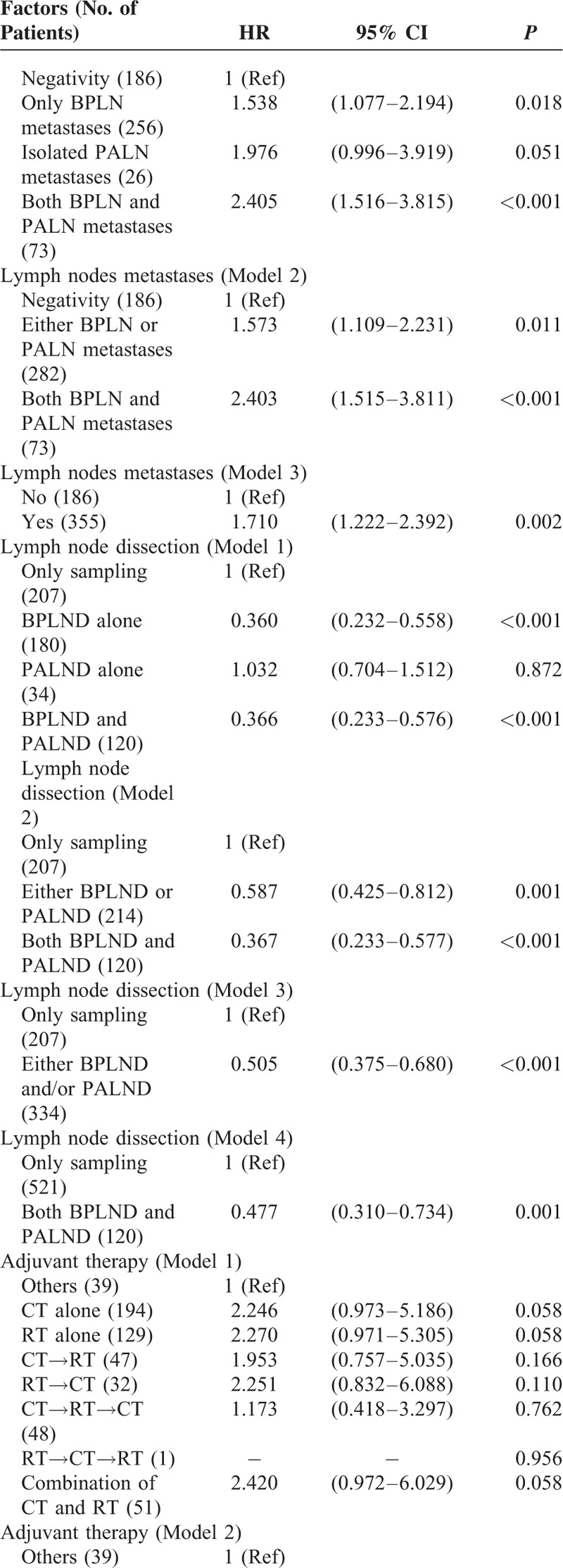
Univariate Analysis for Progress-free Survival

**TABLE 2 (Continued):**
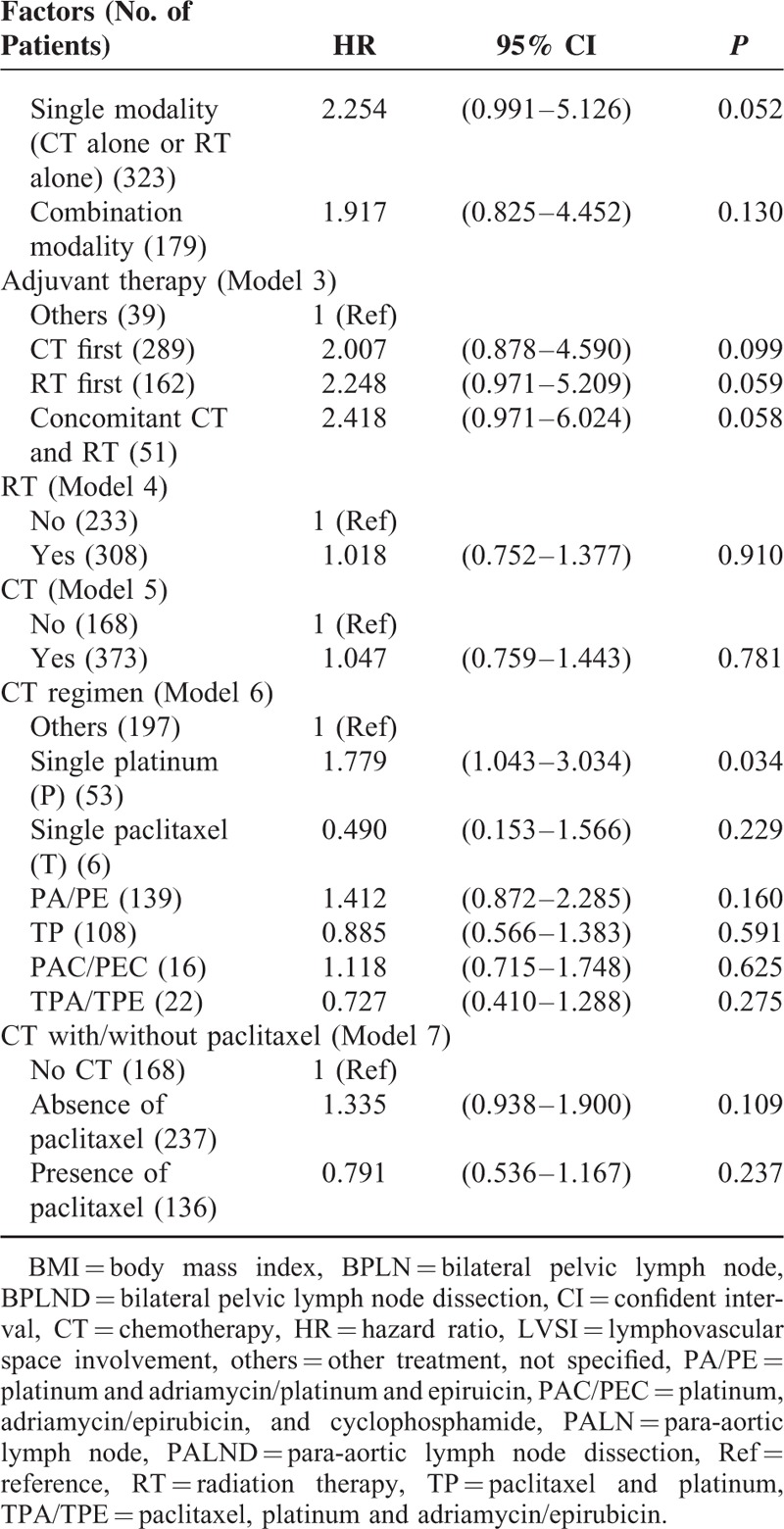
Univariate Analysis for Progress-free Survival

**FIGURE 5:**
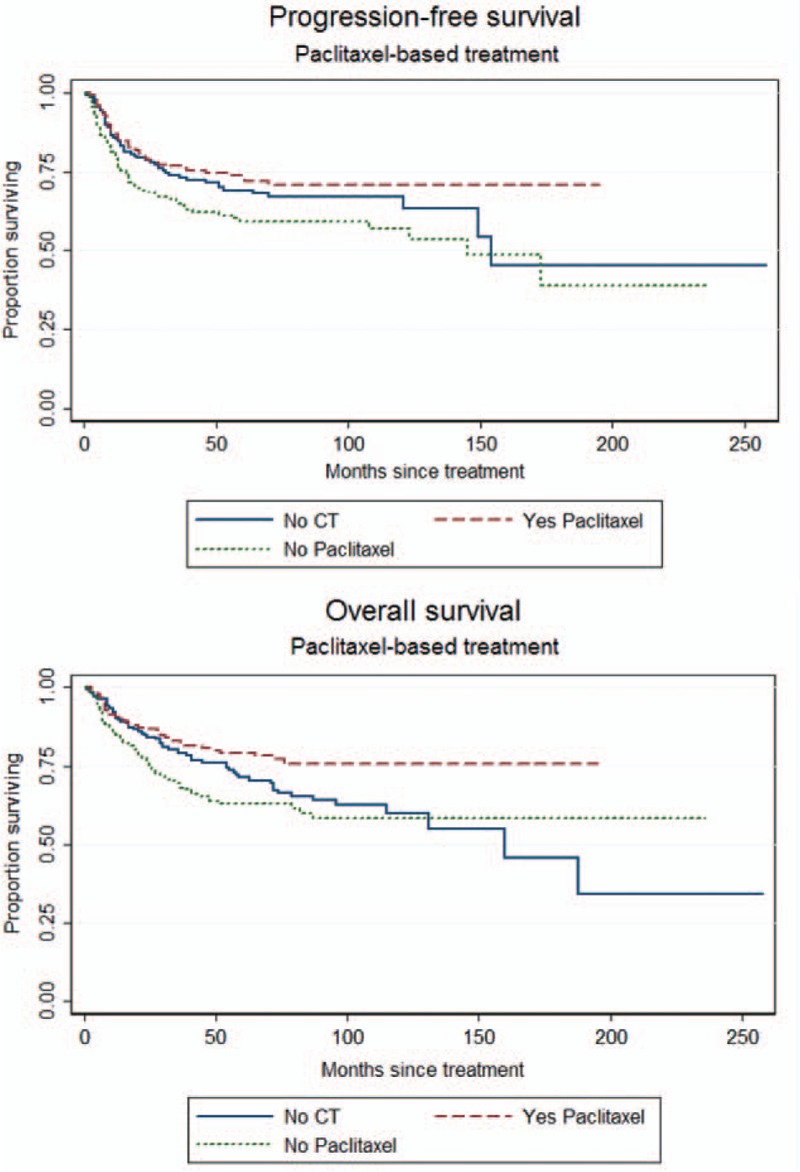
Progression-free survival (PFS) and overall survival (OS) curves according to paclitaxel-based multimodality treatment (No CT: treatment protocol does not contain any chemotherapy; Yes Paclitaxel: paclitaxel-based multi-modality treatment; No Paclitaxel: non-paclitaxel-based multimodality treatment; PFS: log-rank test: *P* = 0.016, OS: log-rank test: *P* = 0.005).

To determine which factors influenced the OS of the patients, univariate analysis showed that age, slim patients, FIGO stage, >1/2 myometrial invasion, histological grade 3, and PLN metastases accompanied with/without PALN metastases contributed to a decrease of OS (Table 3  ). Similar to the findings to PFS, LND significantly increased OS of the patients. Patients who were treated with both LNDs had a best outcome (Figure 4). Although there was no statistical significance among the patients who were treated with different kinds of postoperative adjuvant therapy, paclitaxel-based multimodality treatment seemed to be the best choice because of survival benefits, compared with non-paclitaxel-based multimodality treatment and no CT treatment (Figure 5).

**TABLE 3:**
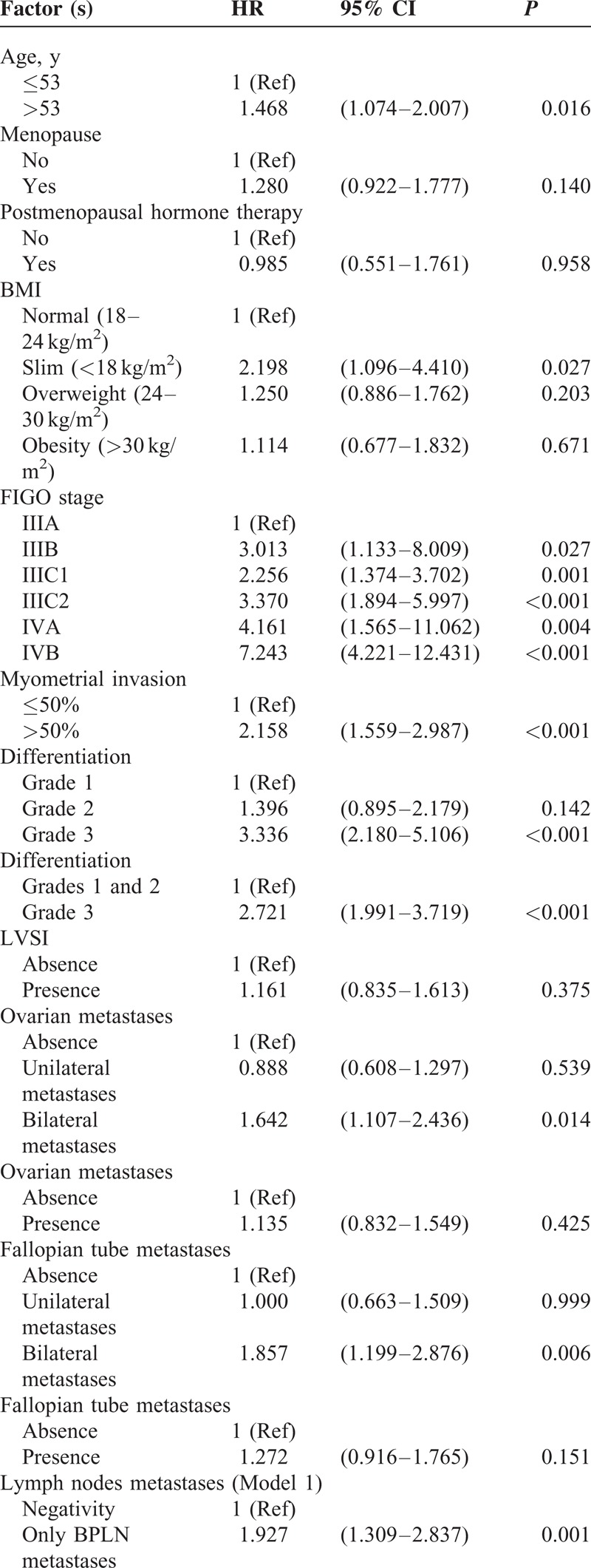
Univariate Analysis for Overall Survival

**TABLE 3 (Continued):**
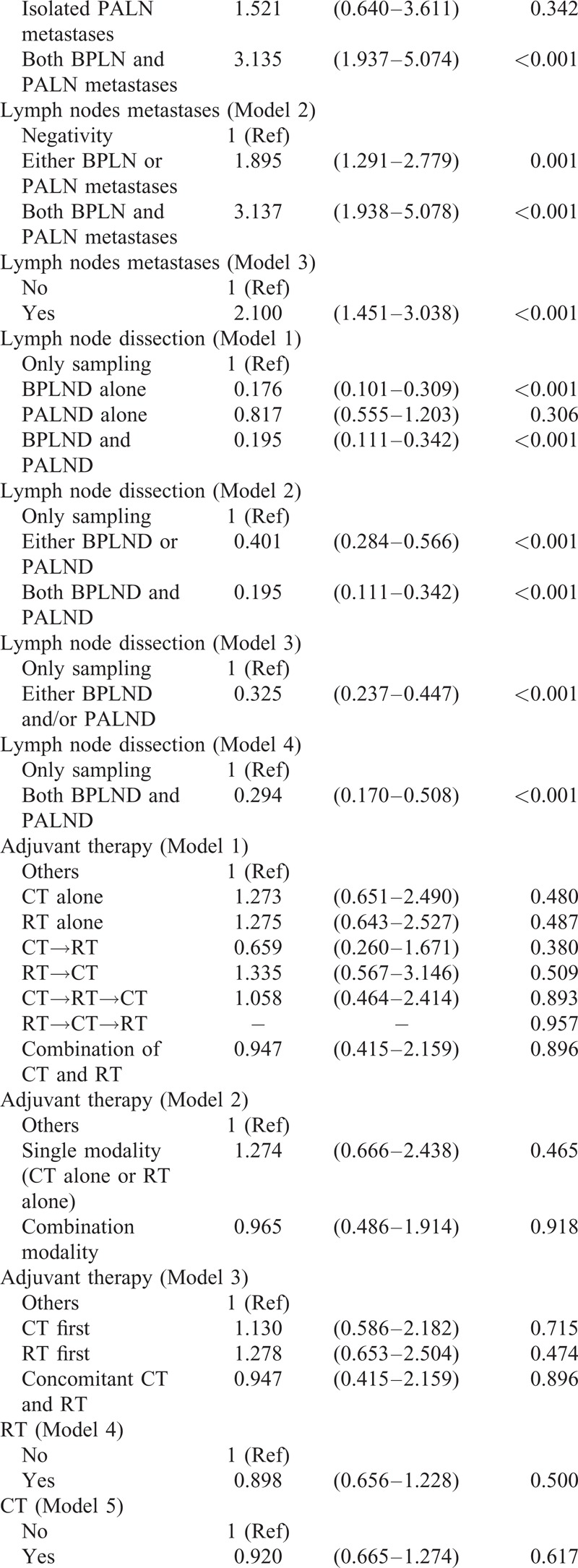
Univariate Analysis for Overall Survival

**TABLE 3 (Continued):**
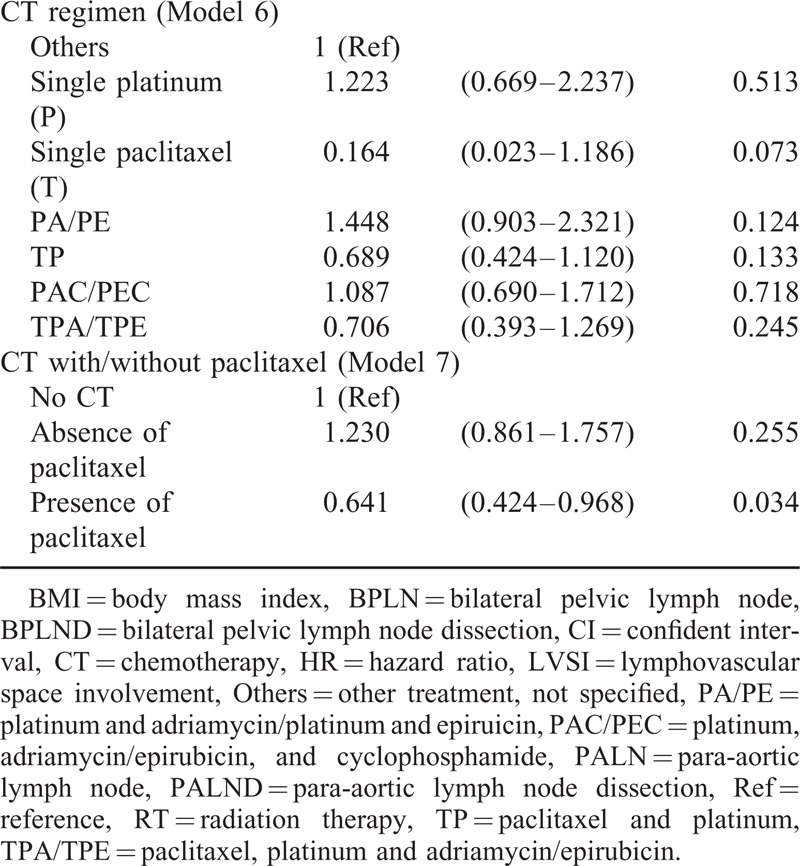
Univariate Analysis for Overall Survival

To further evaluate the impact of above-mentioned parameters on outcome of the patients, multivariate analysis was performed for PFS (Table 4). FIGO stage, >1/2 myometrial invasion, and histological grade 3 were independent risk factors, which significantly decreased the PFS. Besides the above parameters, the modality of the treatment seemed to be associated with outcome. PLND accompanied with/without PALND and postoperative adjuvant therapy containing paclitaxel-based multimodality treatment significantly improved PFS of the patients.

**TABLE 4:**
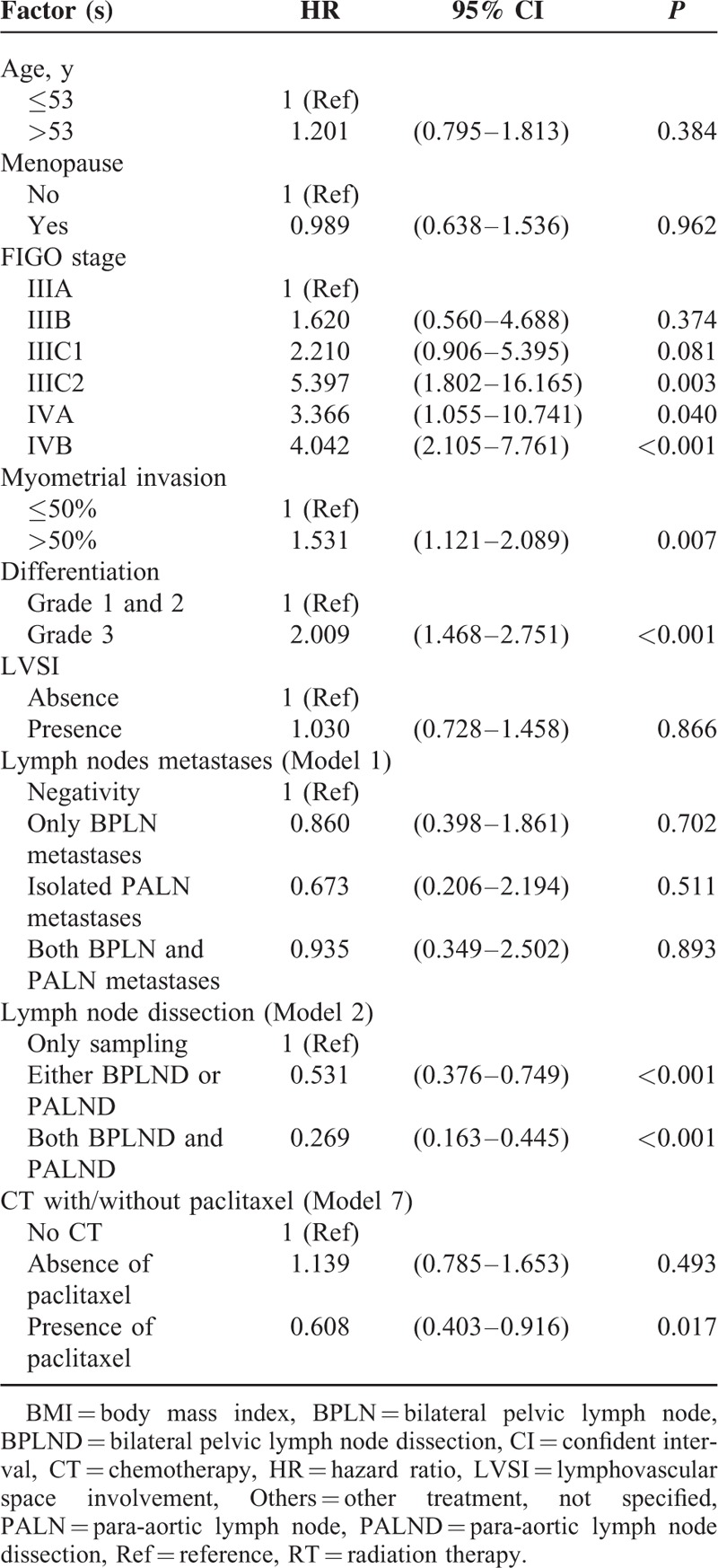
Multivariate Analysis for Progression-free Survival

Multivariate analysis showed that patients with FIGO stage IIIC2 and IVB had the worst outcome (HR 3.22 and 4.59, respectively). Other parameters, including >1/2 myometrial invasion (HR 1.89), histological grade 3 (HR 2.42), and metastases of both BPLN and PALN (HR 2.75) all worsened OS significantly (Table 5). An extensive surgical staging surgery (LND was included) provided a best chance of the OS with a 66% and 86% reduction rate to cancer-related mortality by either LND or both LNDs, respectively. Although patients treated with or without CT seemed to have a similar outcome (Model 5), paclitaxel-based multimodality treatment decreased cancer-related mortality significantly (a 52% reduction).

**TABLE 5:**
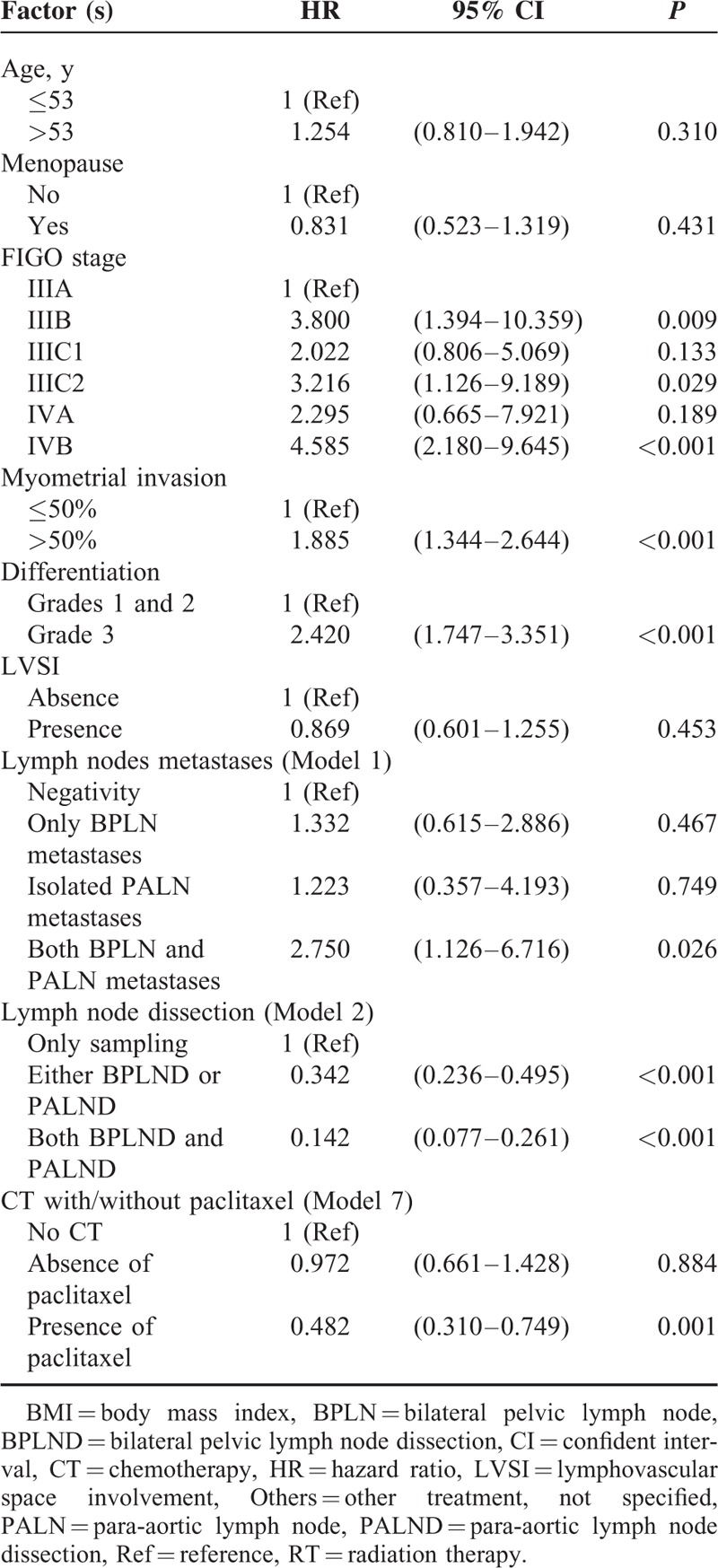
Multivariate Analysis for the Interfered Factors to Overall Survival

## DISCUSSION

Endometrial cancer is generally believed that most patients can be cured and/or controlled adequately by completely surgico-pathological staging surgery.^23^ The main reason is that these patients have their disease detected and treated in the early stage, which was suggested by analysis of the Surveillance, Epidemiology and End Results (SEER) data.^24^ However, data showed that the mortality rate of EC has increased more rapidly than the incidence rate.^25^ There are many possible reasons contributing to increased mortality, including an increased rate of advanced-stage caners, high-risk histological types, such as serous carcinomas, and patients being diagnosed at their advanced age.^25,26^

For advanced-stage EC patients, such as positive LN or stage IV, treatment options at diagnosis are severely limited.^27^ The possible reasons included an inadequate sample size (71 and 162 patients with FIGO stage III E-EC in Mayo Clinic [Rochester, MN] and multiple centers in the United States, respectively),^20^ a significant heterogeneity of study population (134 patients with E-EC in all 356 patients with FIGO III-IV EC, which included high-risk histological types in the multiple centers in USA),^28^ a very heterogeneous group of patients with varying prognostic factors (eg, elder women with EC are less likely to receive surgery, CT, or RT),^29^ and highly variable treatment strategies and CT regimens.^7^

To decrease the influence by these potential confounders, which might influence the treatment plan, including high-risk histological types, such as serous carcinoma, and age factor, our study enrolled the patients who had a complete staging surgery and pathologically confirmed FIGO III-IV E-EC. In addition, the other strengths of the present study included a relatively large number of women (541 patients), the inclusion of many key details, and representative data collection. We believed that the present study might provide useful information to change our policy in the management of these women with FIGO III-IV E-EC, especially for Taiwanese women.

Complete staging surgery might be a key step in the management of women with EC. One of the important basic requirements of complete staging surgery was lymphadenectomy (LND or LNDs). However, the value of lymphadenectomy is still debated, especially for those women with supposed early-stage EC. A recent Cochrane Database Systematic Review, which summarized 3 randomized controlled trials (RCTs) and included 1851 patients, showed no difference of the risk of recurrence or disease-related mortality in presumed stage I disease women either treated with or without lymphadenectomy,^30^ and strongly questioned the benefits of lymphadenectomy for these patients. While some series have demonstrated modest, if any effect on survival for women with early-stage EC who were treated with lymphadenectomy,^31^ others have not, with wide variations in outcome.^32–35^

However, in the management of FIGO III-IV EC, complete resection of tumor might be important; therefore, in theory, lymphadenectomy, especially both LNDs, is an important and key step. Our study supported the necessity of LND as the critical component for successful management of women with FIGO III-IV E-EC. Women undergoing even LND had a significantly better survival than those women undergoing only LNS. Median PFS was increased from 26.0 to 50.5 months significantly. In addition, patients treated with both LNDs had a further increase of median PFS to 58 months. This finding was consistent with a previous small study in Taiwan^36^ and supported both data from the SEPAL (survival effect of para-aortic lymphadenectomy) study^37^ and SEER program.^38^ For stage IIIC-IV patients with LN metastasis, the extent of node resection significantly increased the 5-year disease-specific survival, from 34.4% (1–10 nodes removed) and 62.4% (11–20 nodes removed) to 79.6% (>20 nodes removed) (*P* = 0.04, log-rank test).^36^ For those patients with intermediate or high risk of recurrence in the SEPAL study, procedure including both LNDs could significantly reduce the disease-related mortality rate compared with LND alone (PLND) (HR 0.44, 95% CI 0.30–0.64, *P* < 0.001).^37^ Data from the SEER program showed that the extent of node resection significantly improved the survival from 51.0%, 53.0%, 53.0%, 60.0%, to 72.0% (*P* < .001) for stage IIIC-IV patients with nodal diseases.^38^ The possible mechanisms underlying the survival benefits associated with complete LND included accurate stage and removal of micrometastatic and/or macrometastatic diseases.^39,40^

A high percentage of patients have relatively high incidence of metastases and recurrence and finally die of disease in the advanced-stage EC. Our study showed that FIGO stage, >1/2 myometrial invasion, and histological grade 3 were significantly associated with worst outcome. Physicians need to tailor treatment appropriately to provide the best long-term survival, although there is little agreement about which adjuvant therapy and/or scheduling is the safest and most effective.^10^

To clarify the value of postoperative adjuvant therapy, there are many ongoing trials conducted by the European Network of Gynaecological Oncological Trial Groups (ENGOT) and Gynecologic Oncology Group (GOG) trials, such as ENGOT-EN2-DGCG/EORTC 55102, GOG 249, GOG 258, etc.^30^ There were 8 main types of postoperative adjuvant therapies available in our study, and some of them will be tested by above-mentioned trials. We used 7 models to test what might be better for these patients. However, nearly all models, including various kinds of scheduled treatment (Model 1); single and multiple modality (Model 2); different initiation of treatment, such as CT and RT (Model 3); RT-based CT and CT-based RT (Models 4 and 5); and different CT regimens (Model 6) did not show any statistically significant difference. However, in the model 7, we found that paclitaxel-based multiple modality treatment might provide a better advantage for the patients. As shown in Figure 5, patients undergoing postoperative paclitaxel-based multiple modality treatment might have marginal benefit on PFS and definite benefit on OS. The median PFS was 50 months in the paclitaxel-based multimodality treatment group compared with 34.5 months in the nonpaclitaxel-based multimodality treatment. The median OS was significantly longer in the paclitaxel-based multimodality treatment group than that in the non-paclitaxel multimodality treatment group (56 vs 41 months, *P* = 0.005). Multivariate analysis further confirmed the therapeutic advantages of patients undergoing paclitaxel-based multiple modality treatment with HR of 0.61 (95% CI 0.40–0.92, *P* = 0.017) for PFS and HR of 0.482 (95% CI 0.31–0.75, *P* = 0.001) for OS (Tables 4 and 5).

The limitations of the present study included the selection bias, its retrospective nature, lack of the standardization or details on the treatment, especially for dosage of the CT, and other unspecified classification, and data on management of women with recurrent diseases. The selection bias was mainly based on enrolled criteria, as women who did not undergo a completely surgicopathological staging and/or debulking surgery were excluded in the present study. We did not know the outcome of those patients with advanced-stage E-EC without surgery and those who were treated by incomplete surgery. In addition, there were so limited number of stage IIIB (n = 15) and IVA (n = 10) patients; therefore, the interpretation of results from these subgroups should be careful. Furthermore, the patients were enrolled from many hospitals and study period was long (≥20 years), and all may increase the heterogeneity of study population in the present study. Moreover, we did not further analyze the impact of the different modes of RT, such as external beam and/or vaginal brachytherapy in the present study, which is worthy discussion in a multidisciplinary setting.^21,41^ Last, we did not know the severity and incidence of the side effects and/or adverse events occurred in these patients after treatment. We believed that combination of multimodality therapy and extensive surgery would increase morbidity of these patients, but the use of paclitaxel-based therapy might be a better choice compared with other regimen because of less toxicity.^18^

In conclusion, our study reconfirmed some pathological factors, including >1/2 myometrial invasion, histological grade 3, and FIGO stage were critical factors contributing to worse prognosis of patients with EC, and the phenomenon persisted from early stage to advanced stage of EC. With comprehensive staging surgery, especially a complete resection of the tumor, including both LNDs, the patients might have a better chance to survive. Finally, it was necessary to apply paclitaxel-based multimodality treatment for these patients after complete staging surgery to obtain the better disease control and decrease the cancer-related mortality.
